# Geriatric assessment domains as predictors for clinical endpoints in older adults with cancer: Protocol for an updated systematic review

**DOI:** 10.1371/journal.pone.0319943

**Published:** 2025-03-25

**Authors:** Schroder Sattar, Efthymios Papadopoulos, Kristen R. Haase, Cara Bradley, Caroline Mariano, Isabel Tejero, Rana Jin, Martine Puts, Shabbir M. H. Alibhai

**Affiliations:** 1 College of Nursing, University of Saskatchewan, Saskatoon, Saskatchewan, Canada; 2 School of Kinesiology, College of Human Sciences & Education, Louisiana State University, Baton Rouge, Louisiana, United States of America; 3 Faculty of Applied Science, School of Nursing, University of British Columbia, Vancouver, British Columbia, Canada; 4 BC Cancer Research Institute, Vancouver, British Columbia, Canada; 5 Library, University of Regina, Regina, Saskatchewan, Canada; 6 Department of Geriatric Medicine, Hospital del Mar, Barcelona, Spain; 7 Department of Supportive Care, Princess Margaret Cancer Centre, University Health Network, Toronto, Ontario, Canada; 8 Lawrence Bloomberg Faculty of Nursing, University of Toronto, Toronto, Ontario, Canada; 9 Department of Medicine, University of Toronto, Toronto, Ontario, Canada; 10 Institute of Health Policy, Management, and Evaluation, University of Toronto, Toronto, Ontario, Canada; University of Toronto, CANADA

## Abstract

Geriatric assessments (GA) are increasingly used to inform treatment decision making and tailoring supportive care for older adults with cancer. Identifying which domains predict clinically relevant outcomes might be particularly useful for risk stratification in settings where a GA is not available and/or feasible. The objective of this updated systematic review is to evaluate individual GA domains as predictors for mortality and treatment-related outcomes. Eligible studies will be identified using a predefined search strategy developed in collaboration with an expert librarian in electronic databases (Medline, Cochrane, Embase, CINAHL) and comprise peer-reviewed papers published in any language from July 2017 and reporting on the prospective association between individual GA domains and mortality as well as surgical- or systemic treatment-related outcomes in older adults with cancer. All title/abstract screening, full-text screening, and data extraction will be performed independently by at least 2 authors. Information on cut-offs of GA domains will also be extracted to assess for variability across studies. A decision on performing a meta-analysis versus a narrative summary will be made based on predetermined criteria, which will include heterogeneity among studies and variability in GA tools and cutoff used for each individual domain, as well as level of risk of bias. If a meta-analysis is indicated, a random effects meta-analysis will be conducted using the Comprehensive Meta-Analysis software. The review will be reported in accordance with the Preferred Reporting Items for Systematic Reviews and Meta-analyses (PRISMA) guidelines. This protocol has been registered with PROSPERO (ID: CRD42024580404). This review seeks to investigate individual GA domains as predictors for patient- and treatment-related outcomes. Findings may inform efforts on optimizing GA for this population.

## Introduction

Geriatric assessment (GA) offers substantial benefits for informing oncologic treatment, with alterations in treatment plans for a median 31% of patients; particularly when conducted by multidisciplinary teams [1]. Additionally, GA recommendations can lead to downstream management strategies in over 70% of cases, fostering improved communication between patient and healthcare teams, lower toxicity rates, increased treatment completion rates, and enhanced physical functioning and quality of life [1,2]. Prior meta-analysis has also found an association between comprehensive geriatric assessment (CGA) and a reduction in the incidence of Grade 3+ toxicity [3,4]. However, a full GA is not always feasible due to time constraints (may require up to 60–120 minutes), lack of expertise (e.g., geriatric support) and/or resources. Identifying which domains predict clinically relevant outcomes might be particularly useful for risk stratification in settings where a GA is not available and/or feasible. Various measurement tools exist for assessing geriatric domains, yet no single tool has been found to be superior compared to others. Moreover, the variations in the cutoff scores for delineating impairment among the tools also render inter-study comparisons challenging. A recent retrospective study of 736 older patients found an abnormal GA may be best defined as exhibiting abnormalities in cognition, comorbidities, and having falls risk; with numerical value of 4 (out of the eight domains) being the optimal threshold to predict treatment plan modification [5]. Hence, achieving uniformity in GA is crucial, which in turn necessitates an understanding of the predictive value of each domain to determine inclusion [6].

A prior systematic review by Bruijnen et al. (published in 2019) [6] of 46 studies published between 2006 and 2017 evaluated eight domains (i.e., functional status, cognition, mood, physical function, falls, nutritional status, fatigue, and social support) in the context of geriatric assessment on their predictive ability on various outcomes, including mortality, systemic treatment-related outcomes [e.g., toxicity, completion of planned treatment, and dose modifications], and postoperative complications of elective surgery in the solid tumor setting. The review found all eight domains were predictive for at least one of the investigated outcomes. Of the various domains, both physical function [measured via grip strength or physical performance (e.g., Timed-Up and Go)] and nutritional status were most often associated with systemic treatment-related outcomes and mortality. Altered physical function was also most often associated with postoperative complications. Since this last comprehensive review on this topic by Bruijnen et al. (last search dated as 2017), there has been a significant gap in updating the evidence base surrounding the individual domains of GA on the specific topic. The prior review revealed considerable heterogeneity in findings and identified gaps in the literature, such as the under-examination of certain GA domains (e.g., falls). Given the continued lack of consensus on which domains should be included in GA, it is timely and valuable to conduct an updated systematic review. The reporting of findings by Bruijnen et al. may have impacted subsequent research and attempts to resolve the question over the past several years. Therefore, the objective of this review is to continue to investigate this specific topic by reviewing studies published since the previous review. This review and planned meta-analysis will not only capture new studies published in the past eight years but also evaluate whether recent evidence might refine or change previous conclusions, particularly in the measurement and standardization of GA domain, with the goal of improving the knowledge base on this topic.

In particular, evidence from this systematic review will enhance the understanding of how each GA domain in predicting outcomes in response to surgery or chemotherapy for older adults with cancer, which may be particularly useful in settings where a GA is not available and/or feasible due to time constrains. Our systematic review of newer studies will reveal if efforts have been made in recent studies to address the aforementioned gaps, but also ongoing gaps in the literature to update insights on whether each GA domain predicts outcomes in this population.

## Methods

This protocol has been written in accordance with the Preferred Reporting Items for Systematic Review and Meta-Analysis Protocols (PRISMA-P) guidelines [7], and has been registered with PROSPERO (ID: CRD42024580404). Important protocol amendments will be documented on PROSPERO as well as reported in the paper reporting review findings with clearly justified rationales. See S1 for PRISMA-P Checklist.

### Data sources and search strategy

The search strategy will be developed in collaboration with an experienced university librarian while drawing upon insights from prior systematic reviews on the topic [6]. The search will be conducted by the expert librarian in Medline, Cochrane, Embase, CINAHL from July 2017 (last search in Bruijnen et al. review). The search results will be exported to Covidence, where duplicates will be removed. Data collection has not yet commenced. See S2 for Medline search strategies.

### Study selection

#### Inclusion criteria.

- Studies reporting on patients aged ≥ 65 diagnosed with solid tumors or hematological malignancies of any types and stages, except non-melanoma skin cancer; AND
- Prospective studies investigating GA domains, within the context of baseline GA, as predictors for all- cause and cancer mortality and clinical (i.e., surgical- or systemic treatment-related) outcomes
- No language limit

* Apart from conventional GA domains, measures of muscle strength and physical performance that were included as part of the GA in a study will also constitute eligible outcomes.

#### Exclusion criteria.

- Case studies
- Editorials/opinion papers
- Reviews
- Dissertation thesis
- Abstracts/studies without published full text
- Studies that did not involve performing GA
- Studies exclusively examining association between comorbidity and patient/treatment-related outcomes (In keeping with protocol from Bruijnen et al. [6])

### Operational definitions

Predictor: We will include GA domains that are commonly recommended and assessed in the realm of geriatric assessment and have been reported to have an association with patient outcomes in the oncology setting, and include: functional status, nutrition, cognition, mood, polypharmacy, sensory function, physical function, falls, fatigue, pain, and social supports [1,6,8,9]. For a geriatric domain to constitute a predictor, it must have been assessed at baseline of a prospective study.

For the purposes of this review, our pre-specified outcomes are defined as follows:

- Mortality
  - Cancer-specific mortality and all-cause mortality
- Surgical- and systemic treatment-related outcomes:
  - Grade 3+ toxicity of systemic treatment
  - dose-limiting treatment toxicities
  - completion of planned treatment
  - treatment modifications
  - postoperative complications (grade 3+) for solid tumors
  - ICU admission
  - 30-day postoperative emergency department/hospital readmission
  - Postoperative length of stay

### Other notes

- In keeping with the prior review by Bruijnen et al., for the purposes of this review, comorbidity will not be considered as part of the GA as it is a routine part of the oncological work-up [6].
- The reporting will follow the updated PRISMA 2020 guideline for reporting systematic reviews [10].

### Titles/abstracts screenings and full-text screenings

Titles/abstracts screenings and full-text screenings will be performed independently by two authors. Disagreements will be resolved by discussion. If needed, a third reviewer will be consulted. If two articles reporting on the same study are identified, the one providing more information will be included. The Covidence software will be used to manage the screening and review process. In addition to the reference lists of the included articles, references from the Bruijnen review and other recent systematic reviews will be hand-searched to identify any relevant studies. If a full article cannot be located, efforts will first be made to contact the corresponding author of the abstract. If the corresponding author is unreachable or does not provide a response after the 2^nd^ email, the said abstract will be excluded.

### Data extraction

Data extraction will include study characteristics, including study design, sample size, mean/median age, % female, tumor type, treatment status, geriatric domains access and how measured, and clinical endpoints assessed (that pertain to our pre-specified outcomes of interest (i.e., patient-related outcomes and surgical- and systemic treatment-related outcomes), as specified in previous section. Information on cut-offs of GA domains will also be extracted to assess for variability across studies.

Additionally, information on association between geriatric assessment domains with our outcomes of interests will be extracted. Specifically, study findings on univariate analysis, and whether findings were significant, will be extracted. Additionally, AUC or similar predictive performance measures (e.g., sensitivity, specificity, positive predictive value, negative predictive value) will be extracted to assess the ability of individual GA domains to predict outcomes. If unadjusted analysis results are not reported in the paper, we will contact study authors for the information. In the event of other missing details, corresponding authors will be contacted via email to request the information. All data will be extracted independently by at least 2 authors using a standardized Microsoft Excel sheet. Disagreements will be resolved by discussion. If needed, a third reviewer will be consulted.

### Risk of bias assessment

Risk of bias will be assessed independently by two reviewers using:

- Cochrane Risk of Bias tool (RoB 2) for RCTs [11]
- ROBINS-I for non-randomized studies [12]

### Decision making for meta-analysis

After completing relevant data extraction from the newly identified studies, we will integrate the findings by utilizing the data already extracted in the Bruijnen review, prior to decision-making on meta-analysis. A decision on whether to perform meta-analysis or to summarize and narrate the results regarding predictive ability of individual geriatric domains per outcome measure will be made after data extraction and risk of bias assessment are complete. This decision will be made by expert consensus within the review team, utilizing predetermined criteria; specifically, we will consider factors including heterogeneity between studies, including study designs; variability in GA tools (for each domain) and cutoff points used; diversity of outcomes examined; as well as level of risk of bias.

### Criteria for meta-analysis for a given outcome

- For an outcome to be eligible for meta-analysis, it must be reported in ≥5 eligible studies [13].
- Additionally, if an outcome is reported in ≥10 eligible studies, sensitivity analyses will be conducted by excluding small studies (n < 100) and non-RCTs. Subgroup analyses will also be conducted based on treatment setting (i.e., surgical vs. systemic).
- To prevent double counting, we will ensure samples from each study does not overlap with samples already included in another study. In the event that overlap occurs, data from the largest sample will be used.

### Meta-analysis

Studies will be weighted using the inverse of the variance, based on the DerSimonian and Laird approach [14]. The meta-analysis will be conducted using the Comprehensive Meta Analysis version 3 (Biostat Inc., USA). The overall effect sizes will be reported as mean differences (MD), odds ratios (OR), or hazard ratios (HR) with 95% confidence intervals (CI). Studies with a high RoB will be included in the main analysis. Sensitivity analyses will be conducted to assess the impact of these studies on the overall findings, particularly in the context of high heterogeneity. If their inclusion substantially alters the results, this will be clearly reported and discussed.

#### Dichotomous outcomes.

- Random-effects models with τ² plus Hartung-Knapp adjustments: we will use random-effects models to account for variability between studies. τ² statistic will estimate between-study variance, and Hartung-Knapp adjustments to enhance prevision of our confidence intervals for effect estimates.

#### Continuous outcomes.

- Random-effects models with restricted maximum likelihood estimator of τ², plus Hartung-Knapp adjustments: we will use random-effects models with restricted maximum likelihood estimator to calculate the τ² estimate of between-study variance and apply the Hartung-Knapp adjustments for improved accuracy.

Subgroup analyses will also be conducted based on treatment setting (i.e., surgical vs. systemic).

#### Pre-specified subgroup analysis.

- Treatment setting (surgical vs. systemic)

#### Pre-specified sensitivity analysis.

Exclude:

- Small studies (n < 100)
- Non-RCTs
- Studies with high overall RoB.

#### Handling of heterogeneity.

I^2^ statistic will be used to assess heterogeneity across studies:

- 25% - low heterogeneity
- 50% - moderate heterogeneity
- 75% - high heterogeneity [15]

#### Publication bias.

Funnel plots will be used to assess publication bias if >=10 comparisons are made in a particular meta-analysis. Specifically, funnel plot asymmetry will be assessed by:

- Visual inspection
- Egger test (continuous outcomes); Harbord test (dichotomous outcomes).

#### Certainty of evidence.

Certainty of evidence will be assessed using Grading of Recommendations, Assessment, Development, and Evaluation (GRADE) guidelines and will be assessed independently by 2 authors.

### Narrative synthesis

In the event where meta-analysis is not feasible, a narrative synthesis will be conducted to summarize findings from the included studies. This will include quality assessment and comparison of results of included studies as well as with findings from previous review, as with the case in the Bruijnen et al. [6] paper.

## Discussion

This review will systematically summarize the latest evidence on the predictive ability of each GA domain on clinically relevant outcomes among older adults with cancer. The anticipated significance of this review lies in its potential to streamline and optimize GA in oncology settings by identifying domains that could predict patient- and treatment-related outcomes, aiming to aid clinicians with treatment decisions to optimize the care for older patients. The strengths of this review include its comprehensive approach, which has no language restrictions, and the searches are conducted by an expert research librarian. The search strategies were informed by a prior review on the topic, ensuring a thorough and updated exploration of the literature. Additionally, screening process is rigorous and is conducted independently by at least two reviewers for each item, enhancing the reliability of the findings. However, this review will be based on published studies, which may inadvertently exclude relevant unpublished data, which in turn can limit the comprehensiveness of the review. Additionally, potential variability in how geriatric domains were measured and reported across studies may impact the consistency of findings.

## Conclusion

Findings from this review will extend the knowledge base and understanding of how best to optimize GA in the context of compressed clinic schedule and has potential to promote and guide oncology clinicians in implementing GA.

## Supporting information

S1 File PRISMA-P checklist. (DOCX)

S2 File Medline search strategies. (DOCX)

## References

[1] Hamaker M, Lund C, Te Molder M, Soubeyran P, Wildiers H, van Huis L, et al. Geriatric assessment in the management of older patients with cancer - A systematic review (update). J Geriatr Oncol. 2022;13(6):761–77. doi: 10.1016/j.jgo.2022.04.008 35545495

[2] Mohile SG, Epstein RM, Hurria A, Heckler CE, Canin B, Culakova E, et al. Communication with older patients with cancer using geriatric assessment: a cluster-randomized clinical trial from the national cancer institute community oncology research program. JAMA Oncol. 2020;6(2):196–204. doi: 10.1001/jamaoncol.2019.4728 31697365 PMC6865234

[3] Chuang M-H, Chen J-Y, Tsai W-W, Lee C-W, Lee M-C, Tseng W-H, et al. Impact of comprehensive geriatric assessment on the risk of adverse events in the older patients receiving anti-cancer therapy: a systematic review and meta-analysis. Age Ageing. 2022;51(7):afac145. doi: 10.1093/ageing/afac145 35776674

[4] Anwar MR, Yeretzian ST, Ayala AP, Matosyan E, Breunis H, Bote K, et al. Effectiveness of geriatric assessment and management in older cancer patients: a systematic review and meta-analysis. J Natl Cancer Inst. 2023;115(12):1483–96. doi: 10.1093/jnci/djad200 37738290

[5] Carrozzi A, Jin R, Monginot S, Puts M, Alibhai S. Defining an abnormal geriatric assessment: which deficits matter most?. Cancers. 2023;15(24):5776. 38136321 10.3390/cancers15245776 PMC10742229

[6] Bruijnen CP, van Harten-Krouwel DG, Koldenhof JJ, Emmelot-Vonk MH, Witteveen PO. Predictive value of each geriatric assessment domain for older patients with cancer: A systematic review. J Geriatr Oncol. 2019;10(6):859–73. doi: 10.1016/j.jgo.2019.02.010 30926250

[7] Moher D, Shamseer L, Clarke M, Ghersi D, Liberati A, Petticrew M, et al. Preferred reporting items for systematic review and meta-analysis protocols (PRISMA-P) 2015 statement. Syst Rev. 2015;4(1):1. doi: 10.1186/2046-4053-4-1 25554246 PMC4320440

[8] Sattar S, Alibhai SMH, Wildiers H, Puts MTE. How to implement a geriatric assessment in your clinical practice. Oncologist. 2014;19(10):1056–68. doi: 10.1634/theoncologist.2014-0180 25187477 PMC4200997

[9] Dale W, Klepin H, Williams G, Alibhai S, Bergerot C, Brintzenhofeszoc K, et al. Practical assessment and management of vulnerabilities in older patients receiving systemic cancer therapy: ASCO guideline update. Journal of Clinical Oncology. 2023;23. 10.1200/JCO.23.00933 PMC12803700 37459573

[10] Page MJ, McKenzie JE, Bossuyt PM, Boutron I, Hoffmann TC, Mulrow CD, et al. The PRISMA 2020 statement: an updated guideline for reporting systematic reviews. BMJ. 2021;372(n71). 10.1136/bmj.n71 PMC8005924 33782057

[11] Nejadghaderi SA, Balibegloo M, Rezaei N. The Cochrane risk of bias assessment tool 2 (RoB 2) versus the original RoB: A perspective on the pros and cons. Health Sci Rep. 2024;7(6):e2165. doi: 10.1002/hsr2.2165 38835932 PMC11147813

[12] Higgins JPT, Morgan RL, Rooney AA, Taylor KW, Thayer KA, Silva RA, et al. A tool to assess risk of bias in non-randomized follow-up studies of exposure effects (ROBINS-E). Environ Int. 2024;186:108602. doi: 10.1016/j.envint.2024.108602 38555664 PMC11098530

[13] Takwoingi Y, Guo B, Riley RD, Deeks JJ. Performance of methods for meta-analysis of diagnostic test accuracy with few studies or sparse data. Stat Methods Med Res. 2017;26(4):1896–911. doi: 10.1177/0962280215592269 26116616 PMC5564999

[14] DerSimonian R, Laird N. Meta-analysis in clinical trials. Control Clin Trials. 1986;7(3):177–88. doi: 10.1016/0197-2456(86)90046-2 3802833

[15] Higgins JPT, Thompson SG. Quantifying heterogeneity in a meta-analysis. Stat Med. 2002;21(11):1539–58. doi: 10.1002/sim.1186 12111919

