# Supplementary material for: Geriatric assessment domains as predictors for clinical endpoints in older adults with cancer: Protocol for an updated systematic review
Source: PLoS One. 2025 Mar 25;20(3):e0319943. doi: 10.1371/journal.pone.0319943 (PMC11936278; doi:10.1371/journal.pone.0319943)
Supplement: S2 File — (DOCX) [file pone.0319943.s002.docx]

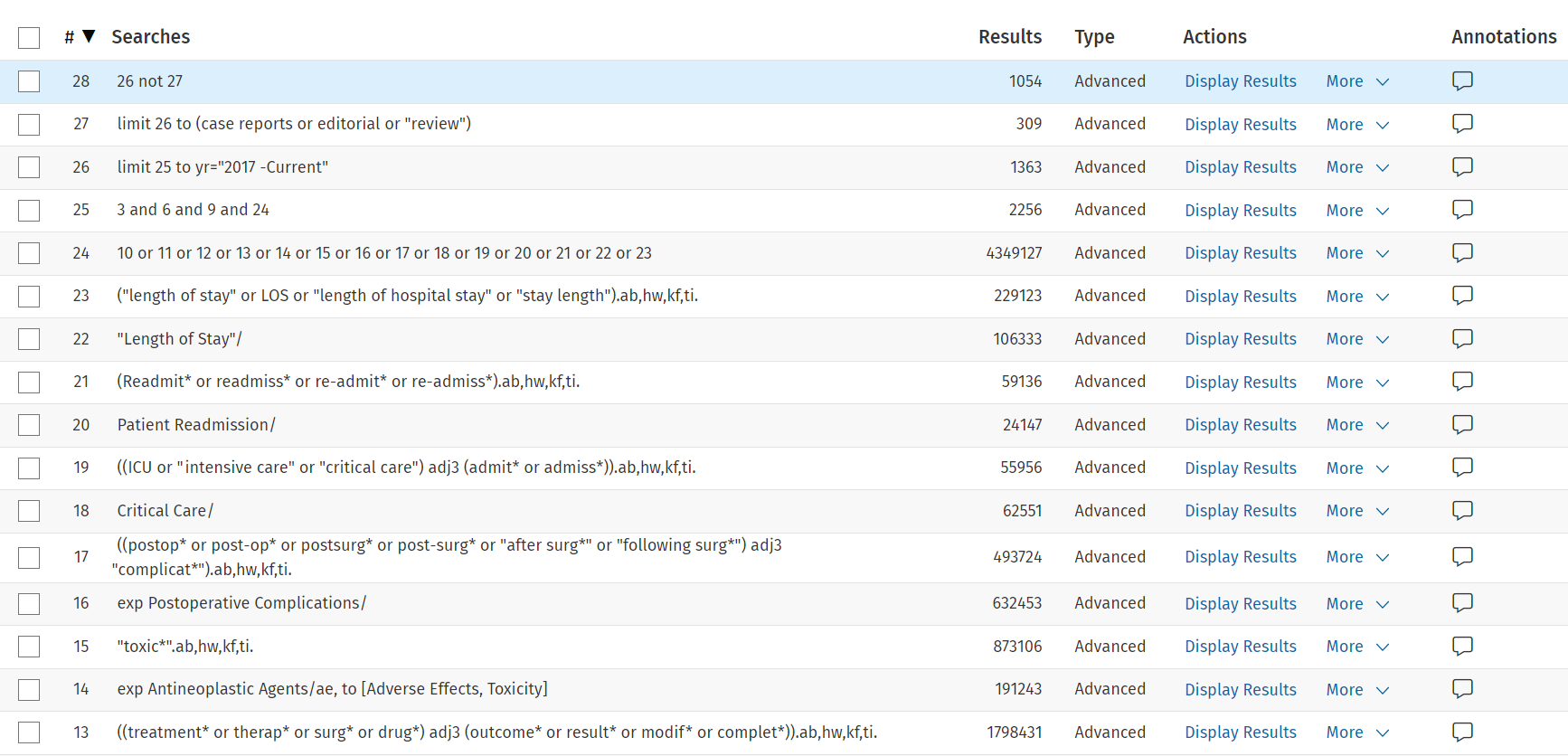


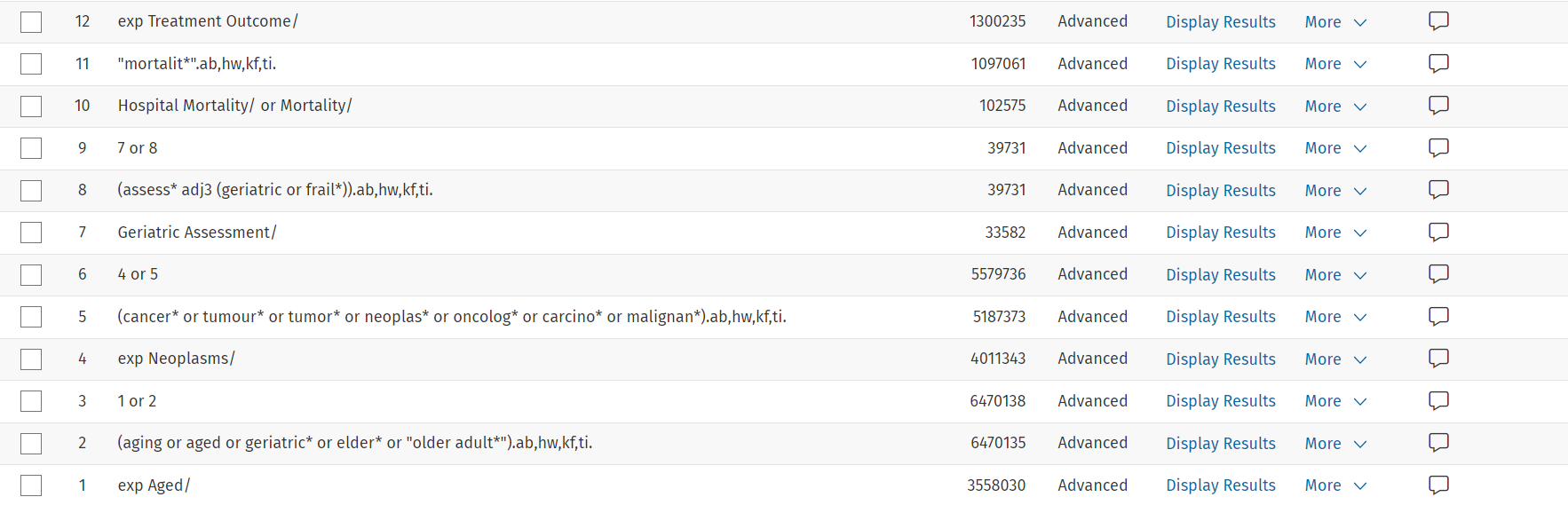


Ovid MEDLINE(R) and In-Process, In-Data-Review & Other Non-Indexed Citations and Daily <1946 to August 29, 2024>

1 exp Aged/ 3558030

2 (aging or aged or geriatric* or elder* or "older adult*").ab,hw,kf,ti. 6470135

3 1 or 2 6470138

4 exp Neoplasms/ 4011343

5 (cancer* or tumour* or tumor* or neoplas* or oncolog* or carcino* or malignan*).ab,hw,kf,ti. 5187373

6 4 or 5 5579736

7 Geriatric Assessment/ 33582

8 (assess* adj3 (geriatric or frail*)).ab,hw,kf,ti. 39731

9 7 or 8 39731

10 Hospital Mortality/ or Mortality/ 102575

11 "mortalit*".ab,hw,kf,ti. 1097061

12 exp Treatment Outcome/ 1300235

13 ((treatment* or therap* or surg* or drug*) adj3 (outcome* or result* or modif* or complet*)).ab,hw,kf,ti. 1798431

14 exp Antineoplastic Agents/ae, to [Adverse Effects, Toxicity] 191243

15 "toxic*".ab,hw,kf,ti. 873106

16 exp Postoperative Complications/ 632453

17 ((postop* or post-op* or postsurg* or post-surg* or "after surg*" or "following surg*") adj3 "complicat*").ab,hw,kf,ti. 493724

18 Critical Care/ 62551

19 ((ICU or "intensive care" or "critical care") adj3 (admit* or admiss*)).ab,hw,kf,ti. 55956

20 Patient Readmission/ 24147

21 (Readmit* or readmiss* or re-admit* or re-admiss*).ab,hw,kf,ti. 59136

22 "Length of Stay"/ 106333

23 ("length of stay" or LOS or "length of hospital stay" or "stay length").ab,hw,kf,ti. 229123

24 10 or 11 or 12 or 13 or 14 or 15 or 16 or 17 or 18 or 19 or 20 or 21 or 22 or 23 4349127

25 3 and 6 and 9 and 24 2256

26 limit 25 to yr="2017 -Current" 1363

27 limit 26 to (case reports or editorial or "review") 309

28 26 not 27 1054
